# Commentary: Differences of Perceived Image Generated through the Web Site: Empirical Evidence Obtained in Spanish Destinations

**DOI:** 10.3389/fpsyg.2020.00735

**Published:** 2020-04-28

**Authors:** Andreas Andronikidis, Victoria Bellou, Nikolaos Stylos, Chris A. Vassiliadis

**Affiliations:** ^1^Department of Business Administration, University of Macedonia, Thessaloniki, Greece; ^2^Department of Economics, University of Thessaly, Volos, Greece; ^3^School of Economics, Finance and Management, University of Bristol, Bristol, United Kingdom

**Keywords:** destination marketing, tourism, perceived images, online content, DMOs

As the investigation of “perceived destination images” and “promotional Web Pages” have been consistent themes in the work of Blazquez-Resino and his colleugues, this commentary is based on the key aspect that using online content posted by DMOs is an important generator of destination images. Based on the view that a destination image is dependent on context, the authors have discussed the impact of analyzing differences of “perceived destination image effects” on the satisfaction and intentions of potential visitors (Blazquez-Resino et al., 2016).

At the beginning of their study, the authors pointed out the leading strategic role that the tourism industry plays in the economic growth and development of numerous countries worldwide. This holds true for Europe as well, where the tourism industry accounts for a substantial contribution to the national economies of the countries across the continent. According to the World Travel & Tourism Council, in 2018, tourism supported 37.4 million jobs and made nearly 2,144 billion USD in contribution to gross domestic product (GDP) or 9.9% of total European GDP (World Travel Tourism Council, 2019). These GDP figures are expected to increase by 2.2% per annum to represent 10.7% of GDP in 2028, a fact that is indicative of the competitive, specialized, and rapidly evolving commercial nature of the tourism industry. It also seems that travel and tourism can be fundamental to the recovery of countries that were hit hard by the recession and the Eurozone crisis, including Greece, Portugal, and Spain. Under these circumstances, tourism sector practitioners are urged to use all available tools and resources in their arsenal to successfully elaborate on the questions of “why” and “how” tourist destinations have an appeal to individuals; and, in turn, they should effectively implement this information to work on their strategies to attract more visitors. Given the importance of tourism to the economic growth of Spain, Blazquez-Resino et al. have investigated in their paper how the Internet could be used as a valuable instrument for marketers to influence future consumer behaviors and attract visitors to specific popular touristic destinations in Spain (Blazquez-Resino et al., 2016). Specifically, they have empirically examined how the information offered by promotional travel websites—Destination Marketing Organizations (DMOs)—shapes individuals' perceived destination image (PDI), which in turn affects their consumer choices via the satisfaction obtained from their internet search results.

To build up their research hypotheses, the authors have used the internet as the main source of information. They particularly focused on the web pages and the internet content of destination marketing organizations. This seems to be an intriguing choice because the distinctive nature of DMOs' websites separates them from other internet content such as travel blogs, commercial and reservation websites, and consumer empowerment sites. More specifically, DMOs (a) are non-profit entities that organize and provide tourism information for a given geographic area and (b) coordinate the local private and public tourism authorities to develop a unique image of the area and lead the overall local tourism industry (Molinillo et al., 2018). This second characteristic of DMOs shows a potential disadvantage of their corresponding websites over other sources of digital information: the different interests and objectives of the different stakeholders involved (both private and public) must ultimately converge to support the marketed image, a fact that is reflected in the quantity, quality, and sophistication of the DMO websites content. The last one could well be the reason for DMO's digital marketing campaigns being challenged by the appearance and popularity of social media applications as well as by the user-generated content (Xiang and Gretzel, 2010; Zeng and Gerritsen, 2014). The evolution of Web 2.0 at the beginning of 2000s, with its user-centric orientation (Anderson, 2016), gave rise to the emergence of travel-related internet content, which is created by individuals and is often termed as tourist generated content (Akehurst, 2009). With the advent of this type of user-generated content, DMOs could not be seen anymore as the most important sources of information search regarding travel destinations. In fact, perceived destination image can be modified by both the information obtained through the official DMO's web-based communication channels (i.e., websites) and the electronic word of mouth (eWOM), which is facilitated by user-generated content (Reza Jalilvand and Samiei, 2012). This way, potential visitors are exposed to two somehow differing streams of digital content; on one hand, DMOs could provide more accurate information on destination location and accessibility, attractive products, service offerings, and community support (Bornhorst et al., 2010). This information is expected to be less biased and of increased reliability and value for consumer choices. At the same time, it could suffer from the need for convergence of all of the different interests and objectives of the stakeholders involved which makes it less diverse. On the other hand, the content (e.g., comments, stories, and experiences) of social media platforms, travel blogs, commercial and reservation websites, and consumer empowerment sites are more personal and varied. Potential visitors may identify more easily with the opinions stated and the stories told (Akehurst, 2009), but these may include both positive and some less favorable comments of questionable credibility and trustworthiness about touristic destinations (Ayeh et al., 2013). The above discussion has highlighted the complexity behind the mechanisms through which present-day tourists form perceptions of travel destinations and offers an interesting avenue for research that might further extend the work of Blazquez-Resino et al. For example, future research may explore how collective memory of differences in promotional web pages and the developed destination image in the mind of potential visitors can be explored using social media data to develop sustainable travel destinations. Furthermore, future scholarly efforts may be directed toward a comparison between the strength of DMOs' and eWOM's differing perspectives (i.e., in terms of digital content) in formulating the online visitors' perceived destination images and associated effects on their consumer choices. This avenue of investigation could be further expanded to include the introduction of and effects emerging from the usage of popular social media platforms by DMOs (e.g., *Facebook, Twitter, Instagram*, and *YouTube)*, which are used as customer services and marketing tools, thus responding to relevant calls from scholars such as Hays et al. (2013). Thus far, there has been little attempt to examine the implementation of social media in DMOs' communication and overall marketing strategies since the majority of studies on social media in the tourism area are consumer-centric. These are mainly focus on travelers' use of social media, though there are a few notable exceptions, e.g., Bigné et al. (2019) and Uşakli et al. (2019).

Overall, we do support the position of Blazquez-Resino and his colleagues in relation to the ability of DMO's digital web-based content to form a favorable image that consumers have on a destination, which would ultimately influence their future consumer behavior. However, it can only be part of the broader picture, where eWOM and social media are treated as significant resources of digital interaction, having the potential to greatly shape future consumer behaviors too, as suggested by Benckendorff et al. (2019). Besides, identifying and understanding the complexities arising by the usage of the available web-based tools (i.e., webpages, eWOM, and social media) is crucial for DMOs in the development of efficient marketing strategies and customer services to motivate and attract individuals from certain target groups of visitors (Katsoni and Venetsanopoulou, 2013; Oliveira and Panyik, 2015). This contemporary tourism context needs to be seen through the lens of wide mobility and the latest developments arising from Web 3.0 architecture, as Ambient Intelligence (AmI) extracts meaning from the way tourists interact with the digital ecosystem (Buhalis, 2019).

## Author Contributions

All authors listed have made a substantial, direct and intellectual contribution to the work, and approved it for publication.

## Conflict of Interest

The authors declare that the research was conducted in the absence of any commercial or financial relationships that could be construed as a potential conflict of interest.
